# *N*-(4-Chloro­phen­yl)-4-meth­oxy-3-(propanamido)­benzamide cyclo­hexane hemisolvate

**DOI:** 10.1107/S1600536812016480

**Published:** 2012-04-21

**Authors:** Zhaojin Zhong, Zhuorong Li, Ningbo Gong, Yanping Li

**Affiliations:** a Institute of Medicinal Biotechnology, Chinese Academy of Medical Sciences and Peking Union Medical College, Beijing 100050, People’s Republic of China; b Institute of Materia Medica, Chinese Academy of Medical Sciences and Peking Union Medical College, Beijing 100050, People’s Republic of China

## Abstract

The title compound, C_17_H_17_ClN_2_O_3_·0.5C_6_H_12_, was prepared by the condensation reaction of 4-meth­oxy-3-(propanamido)­benzoic acid with 4-chloro­aniline. The Cl atom, the propionyl CH_3_ group and the cyclo­hexyl CH_2_ group are disordered over two sets of sites of equal occupancy in both mol­ecules. The cyclo­hexane solvent mol­ecule is disordered over two orientations which were modelled with relative occupancies of 0.484 (4) and 0.516 (4). In the crystal, there are a number of N—H⋯O hydrogen bonds, forming layers perpendicular to (001).

## Related literature

*N*-(4-chloro­phen­yl)-4-meth­oxy-3-(propanamido) benzamide is a lead compound with anti­viral activity targeting APOBEC3G. For the synthesis and properties of the compound and its derivatives, see: Jiang *et al.* (2009 ▶). For APOBEC3G as a target for anti­viral drugs, see: Cullen (2006 ▶); Mamgeat *et al.* (2003 ▶); Cen *et al.*(2004 ▶).
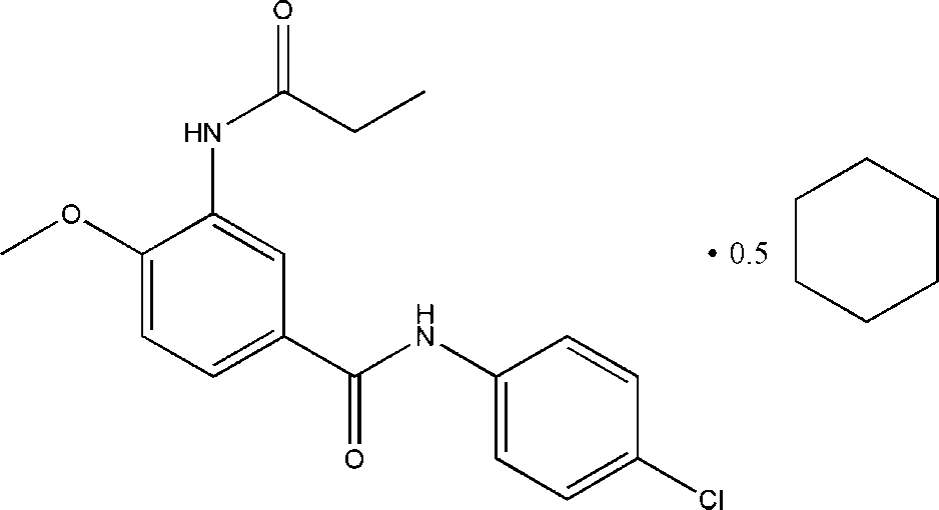


## Experimental

### 

#### Crystal data

C_17_H_17_ClN_2_O_3_·0.5C_6_H_12_
*M* *_r_* = 374.86
Monoclinic, 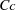

*a* = 16.005 (3) Å
*b* = 18.953 (4) Å
*c* = 14.811 (3) Å
β = 120.08 (3)°
*V* = 3887.8 (18) Å^3^
*Z* = 8
Cu *K*α radiation
μ = 1.92 mm^−1^
*T* = 295 K
0.48 × 0.13 × 0.08 mm

#### Data collection

Rigaku MicroMax 002+ diffractometer
Absorption correction: multi-scan (*REQAB*; Blessing, 1995 ▶) *T*_min_ = 0.743, *T*_max_ = 0.860
22780 measured reflections
5742 independent reflections
5349 reflections with *I* > 2σ(*I*)
*R*_int_ = 0.037

#### Refinement

*R*[*F* ^2^ > 2σ(*F* ^2^)] = 0.044
*wR*(*F* ^2^) = 0.139
*S* = 1.11
5742 reflections
561 parameters
68 restraints
H-atom parameters constrained
Δρ_max_ = 0.23 e Å^−3^
Δρ_min_ = −0.24 e Å^−3^

### 

Data collection: *CrystalClear* (Rigaku, 2008 ▶); cell refinement: *CrystalClear*; data reduction: *CrystalClear*; program(s) used to solve structure: *SHELXS97* (Sheldrick, 2008 ▶); program(s) used to refine structure: *SHELXL97* (Sheldrick, 2008 ▶); molecular graphics: *ORTEPII* (Johnson,1976 ▶) and *PLATON* (Spek, 2009 ▶); software used to prepare material for publication: *SHELXL97*.

## Supplementary Material

Crystal structure: contains datablock(s) I, global. DOI: 10.1107/S1600536812016480/rn2093sup1.cif

Structure factors: contains datablock(s) I. DOI: 10.1107/S1600536812016480/rn2093Isup2.hkl

Supplementary material file. DOI: 10.1107/S1600536812016480/rn2093Isup4.cdx

Supplementary material file. DOI: 10.1107/S1600536812016480/rn2093Isup4.cml

Additional supplementary materials:  crystallographic information; 3D view; checkCIF report

## Figures and Tables

**Table 1:** Hydrogen-bond geometry (Å, °)

*D*—H⋯*A*	*D*—H	H⋯*A*	*D*⋯*A*	*D*—H⋯*A*
N2*A*—H2*AA*⋯O1*B*	0.86	2.25	3.082 (4)	164
N2*B*—H2*BA*⋯O1*A*	0.86	2.26	3.091 (5)	163
N1*A*—H1*AA*⋯O2*B*^i^	0.86	2.28	3.040 (4)	147
N1*B*—H1*BA*⋯O2*A*^ii^	0.86	2.33	3.077 (4)	145

Symmetry codes: (i) 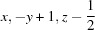; (ii) 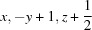.

**Fig. 1.**
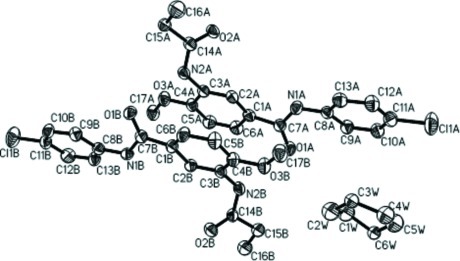
The molecular structure of (I), showing 40% probability displacement ellipsoids and the atom-numbering scheme. Hydrogen atoms have been omitted for clarity.

## supplementary crystallographic information

### Comment

APOBEC3G (or hA3G, human apolipoprotein B mRNA-editing enzyme-catalytic polypeptide-like-3 G) is a target for antiviral drugs (Cullen, 2006; Mamgeat *et al.*, 2003; Cen *et al.*, 2004). In this research area, *N*-(4-chlorophenyl)-4-methoxy-3-(propanamido) benzamide is a lead compound and displays various biological activities (Jiang *et al.*, 2009).

In the molecule of the title compound (I), there are two crystallographically independent benzamide molecules and one cyclohexane molecule in the asymmetric unit. The chlorine, the propionyl CH_3 _and cyclohexyl CH_2_ are disordered. The isotropic displacement parameters of Cl1 (0.1177), Cl2 (0.1153), C16A (0.123) and C16B (0.156) are slightly higher than the average equivalent isotropic displacement parameters (0.065). No restraints were used. The occupancies of Cl1, Cl2, C16A and C16B are all equal to 1. The cyclohexane is disordered having two orientations which have been modelled with relative occupancies of 0.484 (4):0.516 (4). The dihedral angle between the benzene rings in molecule A is 7.86 (25) and this is very similar to that found in molecule B [7.76 (25)]. In the crystal structure, there are a number of N—H···O hydrogen bonds forming layers which are perpendicular to (001). (Table1, Fig. 1).

### Experimental

To a solution of 4-methoxy-3-(propanamido)benzoic acid (10 g, 45 mmol) in *N*,*N*-dimethylformamide (150 ml) was added 1-hydroxybenzotriazole (9 g, 66.6 mmol) and *N*,*N*'-diisopropylcarbodiimide (8.5 ml). After stirring for 30 min at room temperature, *p*-chloroaniline (9 g, 70 mmol) was added. The solution was stirred at room temperature until no starting material was presented by TLC. The crude product was poured into water (200 ml), extracted with dichloromethane (300 ml) and washed with 5% NaOH (100 ml), 5% HCl (100 ml) and water. The organic layer was dried over anhydrous Na_2_SO_4 _and filtered. After the removal of the dichloromethane under reduced pressure, the residue was treated with cyclohexane and filtered. The product, recrystallized with cyclohexane: dichloromethane (1:10), dried *in vacuo* to give *N*-(4-chlorophenyl)-4-methoxy-3-(propanamido) benzamide as a colourless crystalline solid (11.9 g, 79.8%; mp: 423–424 K). ^1^H NMR (CDCl_3_, δ): 8.89 (1*H*, s, –CONH), 8.19 (1*H*, s, 2-H), 7.811 (1*H*, s, –NHCOR), 7.74 (1*H*, d, 6-H), 7.60 (2*H*, d, 2',6'-H), 7.27 (2*H*, d, 3',5'-H), 6.94 (1*H*, d, 5-H), 3.94 (3*H*, s, –OCH_3_), 2.44 (2*H*, q, –CH_2_–), 1.24 (3*H*, t, –CH_3_). MS (EI, *m/z*): 332 (*M*)^+^.

Single crystals suitable for X-ray analysis were obtained by slow evaporation of a mixed solvent of dichloromethane and cyclohexane (3:1 *v/v*).

### Refinement

All H atoms were placed at calculated positions C—H = 0.93–0.97 Å and N—H = 0.86 Å, and were included in the final cycles of refinement using a riding model, with *U*iso(H) = 1.2*U*eq(C,*N*) or 1.5 *U*eq(methyl C). The absolute structure cannot be determined reliably.

### Crystal data

**Table:**

C_17_H_17_ClN_2_O_3_·0.5C_6_H_12_	*F*(000) = 1584
*M**_r_* = 374.86	*D*_x_ = 1.281 Mg m^−^^3^
Monoclinic, *C**c*	Cu *K*α radiation, λ = 1.54178 Å
Hall symbol: C -2yc	Cell parameters from 5349 reflections
*a* = 16.005 (3) Å	θ = 4.1–70.9°
*b* = 18.953 (4) Å	µ = 1.92 mm^−^^1^
*c* = 14.811 (3) Å	*T* = 295 K
β = 120.08 (3)°	Column, colourless
*V* = 3887.8 (18) Å^3^	0.48 × 0.13 × 0.08 mm
*Z* = 8	

### Data collection

**Table:**

Rigaku MicroMax 002+ diffractometer	5742 independent reflections
Confocal monochromator	5349 reflections with *I* > 2σ(*I*)
Detector resolution: 22 pixels mm^-1^	*R*_int_ = 0.037
ω and k scans	θ_max_ = 70.9°, θ_min_ = 4.1°
Absorption correction: multi-scan (REQAB; Blessing, 1995)	*h* = −18→19
*T*_min_ = 0.743, *T*_max_ = 0.860	*k* = −23→22
22780 measured reflections	*l* = −16→15

### Refinement

**Table:**

Refinement on *F*^2^	Secondary atom site location: difference Fourier map
Least-squares matrix: full	Hydrogen site location: inferred from neighbouring sites
*R*[*F*^2^ > 2σ(*F*^2^)] = 0.044	H-atom parameters constrained
*wR*(*F*^2^) = 0.139	*w* = 1/[σ^2^(*F*_o_^2^) + (0.0892*P*)^2^ + 0.5054*P*] where *P* = (*F*_o_^2^ + 2*F*_c_^2^)/3
*S* = 1.11	(Δ/σ)_max_ < 0.001
5742 reflections	Δρ_max_ = 0.23 e Å^−^^3^
561 parameters	Δρ_min_ = −0.24 e Å^−^^3^
68 restraints	Absolute structure: Flack, H. D. (1983). *Acta Cryst.* A39, 876–881.
Primary atom site location: structure-invariant direct methods	Flack parameter: 0.52 (3)

### Special details

**Table:**

Geometry. All e.s.d.'s (except the e.s.d. in the dihedral angle between two l.s. planes) are estimated using the full covariance matrix. The cell e.s.d.'s are taken into account individually in the estimation of e.s.d.'s in distances, angles and torsion angles; correlations between e.s.d.'s in cell parameters are only used when they are defined by crystal symmetry. An approximate (isotropic) treatment of cell e.s.d.'s is used for estimating e.s.d.'s involving l.s. planes.
Refinement. Refinement of *F*^2^ against ALL reflections. The weighted *R*-factor *wR* and goodness of fit *S* are based on *F*^2^, conventional *R*-factors *R* are based on *F*, with *F* set to zero for negative *F*^2^. The threshold expression of *F*^2^ > σ(*F*^2^) is used only for calculating *R*-factors(gt) *etc*. and is not relevant to the choice of reflections for refinement. *R*-factors based on *F*^2^ are statistically about twice as large as those based on *F*, and *R*- factors based on ALL data will be even larger.

### Fractional atomic coordinates and isotropic or equivalent isotropic displacement parameters (Å^2^)

**Table:**

	*x*	*y*	*z*	*U*_iso_*/*U*_eq_	Occ. (<1)
N1A	−0.0625 (2)	0.42524 (17)	0.6331 (3)	0.0569 (7)	
H1AA	−0.0910	0.4607	0.5932	0.068*	
N2A	−0.3579 (2)	0.58212 (16)	0.5663 (3)	0.0571 (7)	
H2AA	−0.3713	0.6071	0.6059	0.069*	
O1A	−0.0714 (2)	0.34408 (13)	0.7413 (2)	0.0687 (7)	
O2A	−0.3531 (2)	0.58003 (15)	0.4173 (2)	0.0694 (7)	
O3A	−0.46711 (19)	0.49448 (16)	0.6056 (3)	0.0667 (7)	
C1A	−0.2055 (2)	0.42062 (18)	0.6501 (3)	0.0531 (8)	
C2A	−0.2391 (2)	0.48788 (18)	0.6127 (3)	0.0505 (7)	
H2AB	−0.2015	0.5178	0.5978	0.061*	
C3A	−0.3273 (2)	0.51154 (16)	0.5969 (3)	0.0497 (7)	
C4A	−0.3843 (2)	0.4658 (2)	0.6200 (3)	0.0545 (8)	
C5A	−0.3520 (3)	0.3984 (2)	0.6541 (3)	0.0610 (9)	
H5AA	−0.3902	0.3681	0.6675	0.073*	
C6A	−0.2669 (3)	0.37518 (19)	0.6687 (4)	0.0597 (9)	
H6AA	−0.2475	0.3291	0.6911	0.072*	
C7A	−0.1080 (2)	0.39287 (19)	0.6780 (3)	0.0523 (8)	
C8A	0.0276 (2)	0.4055 (2)	0.6465 (3)	0.0522 (7)	
C9A	0.0621 (3)	0.3368 (2)	0.6671 (3)	0.0604 (9)	
H9AA	0.0256	0.3018	0.6750	0.072*	
Cl1	0.31464 (10)	0.35026 (10)	0.67527 (16)	0.1177 (6)	
C10A	0.1501 (3)	0.3196 (2)	0.6760 (4)	0.0654 (10)	
H10B	0.1727	0.2734	0.6903	0.078*	
C11A	0.2047 (3)	0.3724 (2)	0.6633 (4)	0.0680 (10)	
C12A	0.1709 (3)	0.4410 (2)	0.6417 (4)	0.0717 (11)	
H12A	0.2069	0.4759	0.6328	0.086*	
C13A	0.0836 (3)	0.4570 (2)	0.6335 (4)	0.0622 (9)	
H13A	0.0611	0.5032	0.6189	0.075*	
C14A	−0.3674 (3)	0.61284 (18)	0.4788 (3)	0.0591 (9)	
C15A	−0.4024 (10)	0.6898 (8)	0.4512 (13)	0.064 (3)	0.484 (12)
H15A	−0.4686	0.6890	0.3939	0.077*	0.484 (12)
H15B	−0.4031	0.7105	0.5107	0.077*	0.484 (12)
C16A	−0.3529 (13)	0.7316 (6)	0.4250 (18)	0.123 (6)	0.484 (12)
H16A	−0.3817	0.7777	0.4093	0.184*	0.484 (12)
H16B	−0.3530	0.7129	0.3647	0.184*	0.484 (12)
H16C	−0.2877	0.7349	0.4818	0.184*	0.484 (12)
C17A	−0.5253 (3)	0.4521 (3)	0.6330 (5)	0.0782 (13)	
H17A	−0.5820	0.4780	0.6194	0.117*	
H17B	−0.4888	0.4403	0.7058	0.117*	
H17C	−0.5440	0.4096	0.5922	0.117*	
C15C	−0.3918 (17)	0.6888 (10)	0.4789 (14)	0.112 (7)	0.516 (12)
H15E	−0.3379	0.7138	0.5350	0.135*	0.516 (12)
H15F	−0.4476	0.6937	0.4879	0.135*	0.516 (12)
C16C	−0.4151 (10)	0.7194 (6)	0.3671 (10)	0.089 (3)	0.516 (12)
H16G	−0.4165	0.7701	0.3685	0.134*	0.516 (12)
H16H	−0.4766	0.7021	0.3139	0.134*	0.516 (12)
H16I	−0.3659	0.7044	0.3523	0.134*	0.516 (12)
N1B	−0.3835 (2)	0.57618 (17)	0.8519 (3)	0.0581 (8)	
H1BA	−0.3542	0.5417	0.8938	0.070*	
N2B	−0.0869 (2)	0.41832 (15)	0.9181 (3)	0.0548 (6)	
H2BA	−0.0717	0.3932	0.8801	0.066*	
O1B	−0.3734 (2)	0.65530 (14)	0.7432 (2)	0.0643 (7)	
O2B	−0.0916 (2)	0.42044 (14)	1.0684 (2)	0.0660 (7)	
O3B	0.0215 (2)	0.50422 (15)	0.8769 (3)	0.0658 (7)	
C1B	−0.2392 (2)	0.58037 (16)	0.8349 (3)	0.0511 (7)	
C2B	−0.2071 (2)	0.51160 (18)	0.8719 (3)	0.0512 (7)	
H2BB	−0.2444	0.4816	0.8870	0.061*	
C3B	−0.1196 (2)	0.48916 (18)	0.8854 (3)	0.0517 (7)	
C4B	−0.0613 (2)	0.53303 (19)	0.8653 (3)	0.0547 (8)	
C5B	−0.0922 (3)	0.6022 (2)	0.8309 (4)	0.0648 (10)	
H5BA	−0.0538	0.6332	0.8188	0.078*	
C6B	−0.1827 (3)	0.62311 (19)	0.8153 (4)	0.0609 (9)	
H6BA	−0.2046	0.6684	0.7904	0.073*	
C7B	−0.3370 (3)	0.60655 (18)	0.8064 (3)	0.0552 (8)	
C8B	−0.4751 (2)	0.5950 (2)	0.8379 (3)	0.0541 (8)	
C9B	−0.5100 (3)	0.66379 (19)	0.8161 (3)	0.0571 (8)	
H9BA	−0.4738	0.6990	0.8079	0.069*	
Cl2	−0.76224 (10)	0.64949 (10)	0.80604 (15)	0.1153 (6)	
C10B	−0.5975 (3)	0.6797 (2)	0.8065 (4)	0.0669 (10)	
H10A	−0.6212	0.7255	0.7913	0.080*	
C11B	−0.6490 (3)	0.6285 (3)	0.8194 (4)	0.0711 (10)	
C12B	−0.6169 (3)	0.5602 (2)	0.8417 (4)	0.0712 (10)	
H12B	−0.6536	0.5258	0.8507	0.085*	
C13B	−0.5291 (3)	0.5433 (2)	0.8506 (4)	0.0657 (10)	
H13B	−0.5064	0.4972	0.8652	0.079*	
C14B	−0.0787 (3)	0.38933 (17)	1.0036 (3)	0.0525 (7)	
C15B	−0.0268 (7)	0.3177 (5)	1.0330 (8)	0.0569 (19)	0.484 (12)
H15C	−0.0371	0.2937	0.9703	0.068*	0.484 (12)
H15D	0.0421	0.3250	1.0777	0.068*	0.484 (12)
C16B	−0.063 (2)	0.2731 (9)	1.088 (2)	0.156 (11)	0.484 (12)
H16D	−0.0295	0.2289	1.1065	0.234*	0.484 (12)
H16E	−0.1312	0.2647	1.0428	0.234*	0.484 (12)
H16F	−0.0533	0.2969	1.1498	0.234*	0.484 (12)
C17B	0.0820 (3)	0.5476 (3)	0.8560 (4)	0.0778 (12)	
H17D	0.1373	0.5211	0.8669	0.117*	
H17E	0.1027	0.5876	0.9021	0.117*	
H17F	0.0468	0.5636	0.7850	0.117*	
C15D	−0.0695 (7)	0.3084 (4)	1.0053 (7)	0.057 (2)	0.516 (12)
H15G	−0.1333	0.2877	0.9650	0.068*	0.516 (12)
H15H	−0.0323	0.2950	0.9727	0.068*	0.516 (12)
C16D	−0.0232 (15)	0.2807 (8)	1.1104 (14)	0.118 (6)	0.516 (12)
H16J	−0.0143	0.2307	1.1087	0.176*	0.516 (12)
H16K	−0.0629	0.2900	1.1406	0.176*	0.516 (12)
H16L	0.0385	0.3031	1.1515	0.176*	0.516 (12)
C1W	0.2002 (11)	0.2975 (8)	0.9773 (14)	0.122 (7)	0.484 (12)
H1WA	0.1604	0.2914	0.9025	0.146*	0.484 (12)
H1WB	0.1662	0.2764	1.0094	0.146*	0.484 (12)
C2W	0.2111 (13)	0.3737 (8)	1.0007 (14)	0.105 (7)	0.484 (12)
H2WA	0.2408	0.3805	1.0755	0.126*	0.484 (12)
H2WB	0.1478	0.3957	0.9678	0.126*	0.484 (12)
C3W	0.2715 (11)	0.4087 (6)	0.963 (2)	0.109 (6)	0.484 (12)
H3WA	0.2358	0.4105	0.8874	0.131*	0.484 (12)
H3WB	0.2851	0.4568	0.9888	0.131*	0.484 (12)
C4W	0.3630 (10)	0.3712 (8)	0.9978 (15)	0.113 (7)	0.484 (12)
H4WA	0.4028	0.3757	1.0730	0.136*	0.484 (12)
H4WB	0.3972	0.3932	0.9667	0.136*	0.484 (12)
C5W	0.3493 (13)	0.2955 (8)	0.970 (2)	0.131 (9)	0.484 (12)
H5WA	0.4118	0.2732	0.9965	0.158*	0.484 (12)
H5WB	0.3143	0.2907	0.8947	0.158*	0.484 (12)
C6W	0.2942 (11)	0.2582 (4)	1.0143 (12)	0.084 (3)	0.484 (12)
H6WA	0.3316	0.2581	1.0900	0.101*	0.484 (12)
H6WB	0.2814	0.2097	0.9903	0.101*	0.484 (12)
C1W'	0.2067 (12)	0.2926 (7)	1.0227 (13)	0.104 (4)	0.516 (12)
H1WC	0.1466	0.2674	0.9992	0.124*	0.516 (12)
H1WD	0.2459	0.2868	1.0977	0.124*	0.516 (12)
C2W'	0.1881 (16)	0.3618 (12)	1.000 (2)	0.143 (8)	0.516 (12)
H2WC	0.1560	0.3802	1.0356	0.172*	0.516 (12)
H2WD	0.1440	0.3668	0.9254	0.172*	0.516 (12)
C3W'	0.2759 (10)	0.4051 (7)	1.0292 (11)	0.091 (4)	0.516 (12)
H3WC	0.3204	0.4012	1.1037	0.109*	0.516 (12)
H3WD	0.2579	0.4543	1.0130	0.109*	0.516 (12)
C4W'	0.3282 (14)	0.3774 (10)	0.9649 (14)	0.112 (7)	0.516 (12)
H4WC	0.2859	0.3839	0.8905	0.135*	0.516 (12)
H4WD	0.3876	0.4032	0.9864	0.135*	0.516 (12)
C5W'	0.3507 (15)	0.2938 (11)	0.993 (3)	0.151 (11)	0.516 (12)
H5WC	0.3754	0.2729	0.9508	0.181*	0.516 (12)
H5WD	0.3985	0.2877	1.0657	0.181*	0.516 (12)
C6W'	0.2609 (14)	0.2609 (8)	0.9694 (12)	0.128 (6)	0.516 (12)
H6WC	0.2737	0.2116	0.9888	0.153*	0.516 (12)
H6WD	0.2178	0.2627	0.8944	0.153*	0.516 (12)

### Atomic displacement parameters (Å^2^)

**Table:**

	*U*^11^	*U*^22^	*U*^33^	*U*^12^	*U*^13^	*U*^23^
N1A	0.0575 (14)	0.0512 (18)	0.070 (2)	0.0041 (11)	0.0384 (12)	0.0116 (12)
N2A	0.0761 (16)	0.0462 (14)	0.0660 (19)	0.0083 (12)	0.0483 (14)	0.0017 (12)
O1A	0.0846 (16)	0.0485 (13)	0.088 (2)	0.0123 (12)	0.0542 (14)	0.0180 (13)
O2A	0.107 (2)	0.0518 (14)	0.0705 (18)	0.0149 (13)	0.0606 (15)	0.0037 (12)
O3A	0.0654 (14)	0.0591 (15)	0.090 (2)	0.0050 (10)	0.0498 (14)	0.0039 (14)
C1A	0.0667 (18)	0.0454 (18)	0.059 (2)	−0.0030 (13)	0.0406 (14)	0.0014 (14)
C2A	0.0595 (16)	0.0430 (15)	0.0573 (19)	−0.0028 (12)	0.0355 (14)	−0.0005 (13)
C3A	0.0651 (17)	0.0362 (14)	0.0558 (19)	−0.0013 (12)	0.0363 (14)	0.0000 (13)
C4A	0.0622 (17)	0.0518 (18)	0.060 (2)	−0.0022 (14)	0.0387 (15)	−0.0016 (14)
C5A	0.071 (2)	0.0449 (16)	0.075 (3)	−0.0065 (15)	0.0428 (18)	0.0072 (16)
C6A	0.074 (2)	0.0410 (15)	0.078 (2)	−0.0112 (14)	0.0484 (18)	0.0012 (15)
C7A	0.0603 (17)	0.0480 (17)	0.059 (2)	0.0034 (13)	0.0374 (15)	0.0039 (14)
C8A	0.0538 (16)	0.0534 (17)	0.0547 (19)	−0.0027 (13)	0.0311 (14)	0.0046 (14)
C9A	0.067 (2)	0.0483 (18)	0.078 (3)	−0.0018 (14)	0.0452 (17)	0.0004 (16)
Cl1	0.0894 (7)	0.1144 (11)	0.1845 (18)	0.0145 (8)	0.0950 (10)	0.0163 (12)
C10A	0.069 (2)	0.057 (2)	0.077 (2)	0.0035 (16)	0.0415 (18)	0.0028 (17)
C11A	0.0633 (19)	0.073 (2)	0.078 (2)	−0.0069 (15)	0.0429 (18)	0.0025 (18)
C12A	0.076 (2)	0.066 (2)	0.092 (3)	−0.0046 (17)	0.056 (2)	0.013 (2)
C13A	0.0729 (19)	0.0485 (19)	0.078 (2)	−0.0050 (15)	0.0475 (18)	0.0087 (17)
C14A	0.085 (2)	0.0374 (16)	0.069 (2)	0.0015 (13)	0.0492 (18)	−0.0011 (14)
C15A	0.113 (6)	0.041 (4)	0.073 (7)	0.018 (4)	0.072 (5)	0.009 (4)
C16A	0.151 (11)	0.048 (4)	0.214 (17)	−0.006 (5)	0.125 (12)	0.001 (6)
C17A	0.070 (2)	0.074 (3)	0.111 (4)	−0.0005 (18)	0.060 (2)	0.005 (2)
C15C	0.243 (18)	0.047 (5)	0.075 (9)	0.025 (6)	0.101 (9)	0.017 (5)
C16C	0.121 (7)	0.055 (5)	0.103 (7)	0.034 (5)	0.065 (6)	0.047 (5)
N1B	0.0671 (16)	0.0512 (18)	0.0622 (19)	0.0120 (12)	0.0372 (13)	0.0144 (12)
N2B	0.0771 (16)	0.0391 (14)	0.0632 (18)	0.0109 (12)	0.0465 (14)	0.0050 (11)
O1B	0.0735 (14)	0.0581 (14)	0.0803 (18)	0.0183 (11)	0.0527 (13)	0.0247 (13)
O2B	0.0976 (18)	0.0465 (13)	0.0721 (17)	0.0102 (11)	0.0561 (14)	0.0042 (11)
O3B	0.0704 (14)	0.0544 (14)	0.091 (2)	0.0045 (11)	0.0542 (14)	0.0065 (13)
C1B	0.0572 (17)	0.0375 (16)	0.060 (2)	0.0019 (11)	0.0309 (13)	0.0015 (13)
C2B	0.0641 (17)	0.0413 (16)	0.059 (2)	0.0009 (13)	0.0385 (15)	0.0046 (13)
C3B	0.0650 (18)	0.0434 (16)	0.055 (2)	0.0069 (13)	0.0360 (15)	0.0027 (13)
C4B	0.0559 (15)	0.0471 (16)	0.066 (2)	0.0020 (13)	0.0342 (14)	−0.0006 (15)
C5B	0.0632 (19)	0.0478 (18)	0.097 (3)	−0.0080 (14)	0.050 (2)	−0.0026 (19)
C6B	0.0647 (18)	0.0395 (15)	0.081 (3)	0.0091 (13)	0.0384 (17)	0.0098 (15)
C7B	0.0698 (19)	0.0379 (15)	0.063 (2)	0.0012 (13)	0.0373 (16)	0.0022 (13)
C8B	0.0640 (18)	0.0489 (17)	0.054 (2)	0.0043 (13)	0.0328 (15)	0.0020 (15)
C9B	0.0649 (18)	0.0458 (17)	0.064 (2)	−0.0014 (14)	0.0349 (15)	0.0002 (15)
Cl2	0.0894 (7)	0.1148 (11)	0.1774 (16)	0.0232 (8)	0.0936 (10)	0.0300 (12)
C10B	0.074 (2)	0.054 (2)	0.084 (3)	0.0139 (16)	0.0480 (19)	0.0060 (18)
C11B	0.071 (2)	0.076 (3)	0.079 (3)	0.0173 (17)	0.0475 (19)	0.0076 (19)
C12B	0.071 (2)	0.066 (2)	0.085 (3)	−0.0087 (17)	0.046 (2)	0.002 (2)
C13B	0.075 (2)	0.053 (2)	0.079 (3)	0.0097 (16)	0.0460 (18)	0.0108 (18)
C14B	0.0629 (17)	0.0419 (16)	0.066 (2)	0.0063 (12)	0.0418 (15)	0.0049 (13)
C15B	0.061 (4)	0.050 (3)	0.065 (5)	0.010 (3)	0.036 (4)	0.011 (3)
C16B	0.23 (3)	0.073 (8)	0.25 (3)	0.045 (11)	0.18 (2)	0.090 (13)
C17B	0.073 (2)	0.080 (3)	0.099 (3)	0.0023 (19)	0.057 (2)	0.013 (2)
C15D	0.057 (4)	0.035 (3)	0.074 (5)	0.009 (3)	0.031 (4)	0.003 (3)
C16D	0.151 (12)	0.075 (7)	0.109 (9)	0.016 (6)	0.052 (7)	−0.013 (6)
C1W	0.127 (9)	0.116 (12)	0.109 (12)	0.002 (8)	0.049 (9)	0.045 (9)
C2W	0.137 (17)	0.073 (8)	0.088 (8)	0.027 (10)	0.043 (9)	0.004 (6)
C3W	0.103 (8)	0.061 (5)	0.161 (17)	0.015 (5)	0.064 (9)	0.001 (7)
C4W	0.082 (9)	0.093 (8)	0.118 (15)	−0.021 (7)	0.016 (8)	0.015 (8)
C5W	0.094 (9)	0.161 (17)	0.148 (16)	−0.025 (9)	0.067 (9)	0.029 (11)
C6W	0.130 (9)	0.038 (3)	0.102 (8)	0.012 (4)	0.070 (7)	0.019 (4)
C1W'	0.132 (9)	0.068 (5)	0.101 (9)	−0.019 (6)	0.052 (7)	−0.007 (5)
C2W'	0.089 (8)	0.127 (13)	0.22 (2)	−0.014 (8)	0.078 (10)	−0.005 (11)
C3W'	0.111 (9)	0.066 (6)	0.070 (5)	0.012 (5)	0.027 (5)	−0.007 (6)
C4W'	0.124 (13)	0.112 (12)	0.086 (9)	−0.052 (12)	0.040 (10)	−0.004 (7)
C5W'	0.132 (13)	0.142 (17)	0.21 (2)	0.079 (13)	0.112 (14)	0.047 (14)
C6W'	0.149 (13)	0.105 (10)	0.081 (8)	0.026 (8)	0.021 (8)	−0.003 (6)

### Geometric parameters (Å, º)

**Table:**

N1A—C7A	1.355 (5)	C5B—H5BA	0.9300
N1A—C8A	1.404 (4)	C6B—H6BA	0.9300
N1A—H1AA	0.8600	C8B—C13B	1.381 (6)
N2A—C14A	1.358 (5)	C8B—C9B	1.391 (5)
N2A—C3A	1.419 (4)	C9B—C10B	1.369 (6)
N2A—H2AA	0.8600	C9B—H9BA	0.9300
O1A—C7A	1.235 (5)	Cl2—C11B	1.767 (4)
O2A—C14A	1.215 (5)	C10B—C11B	1.347 (7)
O3A—C4A	1.347 (4)	C10B—H10A	0.9300
O3A—C17A	1.434 (5)	C11B—C12B	1.371 (7)
C1A—C2A	1.387 (5)	C12B—C13B	1.382 (6)
C1A—C6A	1.433 (4)	C12B—H12B	0.9300
C1A—C7A	1.496 (5)	C13B—H13B	0.9300
C2A—C3A	1.385 (5)	C14B—C15B	1.537 (9)
C2A—H2AB	0.9300	C14B—C15D	1.541 (8)
C3A—C4A	1.419 (5)	C15B—C16B	1.482 (19)
C4A—C5A	1.375 (5)	C15B—H15C	0.9700
C5A—C6A	1.342 (5)	C15B—H15D	0.9700
C5A—H5AA	0.9300	C16B—H16D	0.9600
C6A—H6AA	0.9300	C16B—H16E	0.9600
C8A—C9A	1.387 (5)	C16B—H16F	0.9600
C8A—C13A	1.402 (5)	C17B—H17D	0.9600
C9A—C10A	1.386 (6)	C17B—H17E	0.9600
C9A—H9AA	0.9300	C17B—H17F	0.9600
Cl1—C11A	1.730 (4)	C15D—C16D	1.45 (2)
C10A—C11A	1.401 (6)	C15D—H15G	0.9700
C10A—H10B	0.9300	C15D—H15H	0.9700
C11A—C12A	1.383 (6)	C16D—H16J	0.9600
C12A—C13A	1.373 (6)	C16D—H16K	0.9600
C12A—H12A	0.9300	C16D—H16L	0.9600
C13A—H13A	0.9300	C1W—C2W	1.476 (16)
C14A—C15C	1.492 (19)	C1W—C6W	1.514 (15)
C14A—C15A	1.543 (15)	C1W—H1WA	0.9700
C15A—C16A	1.310 (19)	C1W—H1WB	0.9700
C15A—H15A	0.9700	C2W—C3W	1.493 (16)
C15A—H15B	0.9700	C2W—H2WA	0.9700
C16A—H16A	0.9600	C2W—H2WB	0.9700
C16A—H16B	0.9600	C3W—C4W	1.469 (15)
C16A—H16C	0.9600	C3W—H3WA	0.9700
C17A—H17A	0.9600	C3W—H3WB	0.9700
C17A—H17B	0.9600	C4W—C5W	1.478 (16)
C17A—H17C	0.9600	C4W—H4WA	0.9700
C15C—C16C	1.61 (2)	C4W—H4WB	0.9700
C15C—H15E	0.9700	C5W—C6W	1.513 (15)
C15C—H15F	0.9700	C5W—H5WA	0.9700
C16C—H16G	0.9600	C5W—H5WB	0.9700
C16C—H16H	0.9600	C6W—H6WA	0.9700
C16C—H16I	0.9600	C6W—H6WB	0.9700
N1B—C7B	1.357 (5)	C1W'—C2W'	1.35 (3)
N1B—C8B	1.420 (5)	C1W'—C6W'	1.56 (3)
N1B—H1BA	0.8600	C1W'—H1WC	0.9700
N2B—C14B	1.325 (5)	C1W'—H1WD	0.9700
N2B—C3B	1.434 (4)	C2W'—C3W'	1.49 (2)
N2B—H2BA	0.8600	C2W'—H2WC	0.9700
O1B—C7B	1.234 (5)	C2W'—H2WD	0.9700
O2B—C14B	1.228 (5)	C3W'—C4W'	1.64 (3)
O3B—C4B	1.363 (4)	C3W'—H3WC	0.9700
O3B—C17B	1.417 (5)	C3W'—H3WD	0.9700
C1B—C6B	1.349 (5)	C4W'—C5W'	1.63 (2)
C1B—C2B	1.408 (4)	C4W'—H4WC	0.9700
C1B—C7B	1.487 (5)	C4W'—H4WD	0.9700
C2B—C3B	1.378 (5)	C5W'—C6W'	1.44 (3)
C2B—H2BB	0.9300	C5W'—H5WC	0.9700
C3B—C4B	1.389 (5)	C5W'—H5WD	0.9700
C4B—C5B	1.403 (5)	C6W'—H6WC	0.9700
C5B—C6B	1.405 (5)	C6W'—H6WD	0.9700
			
C7A—N1A—C8A	125.3 (3)	C9B—C10B—H10A	120.2
C7A—N1A—H1AA	117.4	C10B—C11B—C12B	122.1 (4)
C8A—N1A—H1AA	117.4	C10B—C11B—Cl2	119.2 (3)
C14A—N2A—C3A	124.4 (3)	C12B—C11B—Cl2	118.6 (4)
C14A—N2A—H2AA	117.8	C11B—C12B—C13B	118.8 (4)
C3A—N2A—H2AA	117.8	C11B—C12B—H12B	120.6
C4A—O3A—C17A	117.1 (3)	C13B—C12B—H12B	120.6
C2A—C1A—C6A	117.6 (3)	C8B—C13B—C12B	119.9 (4)
C2A—C1A—C7A	125.1 (3)	C8B—C13B—H13B	120.0
C6A—C1A—C7A	117.1 (3)	C12B—C13B—H13B	120.0
C3A—C2A—C1A	121.6 (3)	O2B—C14B—N2B	124.9 (3)
C3A—C2A—H2AB	119.2	O2B—C14B—C15B	118.8 (5)
C1A—C2A—H2AB	119.2	N2B—C14B—C15B	114.7 (5)
C2A—C3A—N2A	121.2 (3)	O2B—C14B—C15D	121.2 (5)
C2A—C3A—C4A	119.1 (3)	N2B—C14B—C15D	113.0 (5)
N2A—C3A—C4A	119.5 (3)	C15B—C14B—C15D	23.3 (3)
O3A—C4A—C5A	126.3 (3)	C16B—C15B—C14B	110.6 (10)
O3A—C4A—C3A	114.5 (3)	C16B—C15B—H15C	109.5
C5A—C4A—C3A	119.1 (3)	C14B—C15B—H15C	109.5
C6A—C5A—C4A	121.9 (3)	C16B—C15B—H15D	109.5
C6A—C5A—H5AA	119.0	C14B—C15B—H15D	109.5
C4A—C5A—H5AA	119.0	H15C—C15B—H15D	108.1
C5A—C6A—C1A	120.6 (3)	C15B—C16B—H16D	109.5
C5A—C6A—H6AA	119.7	C15B—C16B—H16E	109.5
C1A—C6A—H6AA	119.7	H16D—C16B—H16E	109.5
O1A—C7A—N1A	122.9 (3)	C15B—C16B—H16F	109.5
O1A—C7A—C1A	120.4 (3)	H16D—C16B—H16F	109.5
N1A—C7A—C1A	116.7 (3)	H16E—C16B—H16F	109.5
C9A—C8A—C13A	118.2 (3)	O3B—C17B—H17D	109.5
C9A—C8A—N1A	123.2 (3)	O3B—C17B—H17E	109.5
C13A—C8A—N1A	118.5 (3)	H17D—C17B—H17E	109.5
C10A—C9A—C8A	121.0 (3)	O3B—C17B—H17F	109.5
C10A—C9A—H9AA	119.5	H17D—C17B—H17F	109.5
C8A—C9A—H9AA	119.5	H17E—C17B—H17F	109.5
C9A—C10A—C11A	119.4 (4)	C16D—C15D—C14B	111.8 (8)
C9A—C10A—H10B	120.3	C16D—C15D—H15G	109.3
C11A—C10A—H10B	120.3	C14B—C15D—H15G	109.3
C12A—C11A—C10A	120.3 (4)	C16D—C15D—H15H	109.2
C12A—C11A—Cl1	120.7 (3)	C14B—C15D—H15H	109.2
C10A—C11A—Cl1	118.9 (4)	H15G—C15D—H15H	107.9
C13A—C12A—C11A	119.4 (4)	C15D—C16D—H16J	109.5
C13A—C12A—H12A	120.3	C15D—C16D—H16K	109.5
C11A—C12A—H12A	120.3	H16J—C16D—H16K	109.5
C12A—C13A—C8A	121.7 (4)	C15D—C16D—H16L	109.5
C12A—C13A—H13A	119.1	H16J—C16D—H16L	109.5
C8A—C13A—H13A	119.1	H16K—C16D—H16L	109.5
O2A—C14A—N2A	121.5 (3)	C2W—C1W—C6W	114.7 (11)
O2A—C14A—C15C	130.0 (8)	C2W—C1W—H1WA	108.6
N2A—C14A—C15C	108.4 (7)	C6W—C1W—H1WA	108.6
O2A—C14A—C15A	117.7 (7)	C2W—C1W—H1WB	108.6
N2A—C14A—C15A	120.7 (7)	C6W—C1W—H1WB	108.6
C15C—C14A—C15A	13.4 (11)	H1WA—C1W—H1WB	107.6
C16A—C15A—C14A	117.2 (10)	C1W—C2W—C3W	111.7 (11)
C16A—C15A—H15A	108.0	C1W—C2W—H2WA	109.3
C14A—C15A—H15A	108.0	C3W—C2W—H2WA	109.3
C16A—C15A—H15B	108.0	C1W—C2W—H2WB	109.3
C14A—C15A—H15B	108.0	C3W—C2W—H2WB	109.3
H15A—C15A—H15B	107.2	H2WA—C2W—H2WB	107.9
C15A—C16A—H16A	109.5	C4W—C3W—C2W	111.8 (13)
C15A—C16A—H16B	109.5	C4W—C3W—H3WA	109.3
H16A—C16A—H16B	109.5	C2W—C3W—H3WA	109.2
C15A—C16A—H16C	109.5	C4W—C3W—H3WB	109.3
H16A—C16A—H16C	109.5	C2W—C3W—H3WB	109.3
H16B—C16A—H16C	109.5	H3WA—C3W—H3WB	107.9
O3A—C17A—H17A	109.5	C3W—C4W—C5W	112.9 (12)
O3A—C17A—H17B	109.5	C3W—C4W—H4WA	109.0
H17A—C17A—H17B	109.5	C5W—C4W—H4WA	109.0
O3A—C17A—H17C	109.5	C3W—C4W—H4WB	109.0
H17A—C17A—H17C	109.5	C5W—C4W—H4WB	109.0
H17B—C17A—H17C	109.5	H4WA—C4W—H4WB	107.8
C14A—C15C—C16C	105.8 (12)	C4W—C5W—C6W	111.7 (14)
C14A—C15C—H15E	110.6	C4W—C5W—H5WA	109.3
C16C—C15C—H15E	110.6	C6W—C5W—H5WA	109.3
C14A—C15C—H15F	110.6	C4W—C5W—H5WB	109.3
C16C—C15C—H15F	110.6	C6W—C5W—H5WB	109.3
H15E—C15C—H15F	108.7	H5WA—C5W—H5WB	107.9
C15C—C16C—H16G	109.5	C5W—C6W—C1W	107.6 (10)
C15C—C16C—H16H	109.5	C5W—C6W—H6WA	110.2
H16G—C16C—H16H	109.5	C1W—C6W—H6WA	110.2
C15C—C16C—H16I	109.5	C5W—C6W—H6WB	110.2
H16G—C16C—H16I	109.5	C1W—C6W—H6WB	110.2
H16H—C16C—H16I	109.5	H6WA—C6W—H6WB	108.5
C7B—N1B—C8B	127.2 (3)	C2W'—C1W'—C6W'	110.9 (18)
C7B—N1B—H1BA	116.4	C2W'—C1W'—H1WC	109.5
C8B—N1B—H1BA	116.4	C6W'—C1W'—H1WC	109.5
C14B—N2B—C3B	123.7 (3)	C2W'—C1W'—H1WD	109.5
C14B—N2B—H2BA	118.1	C6W'—C1W'—H1WD	109.5
C3B—N2B—H2BA	118.1	H1WC—C1W'—H1WD	108.1
C4B—O3B—C17B	117.9 (3)	C1W'—C2W'—C3W'	113.8 (17)
C6B—C1B—C2B	119.1 (3)	C1W'—C2W'—H2WC	108.8
C6B—C1B—C7B	117.5 (3)	C3W'—C2W'—H2WC	108.8
C2B—C1B—C7B	123.1 (3)	C1W'—C2W'—H2WD	108.8
C3B—C2B—C1B	119.3 (3)	C3W'—C2W'—H2WD	108.8
C3B—C2B—H2BB	120.4	H2WC—C2W'—H2WD	107.7
C1B—C2B—H2BB	120.4	C2W'—C3W'—C4W'	109.6 (13)
C2B—C3B—C4B	121.8 (3)	C2W'—C3W'—H3WC	109.8
C2B—C3B—N2B	120.7 (3)	C4W'—C3W'—H3WC	109.8
C4B—C3B—N2B	117.5 (3)	C2W'—C3W'—H3WD	109.8
O3B—C4B—C3B	116.7 (3)	C4W'—C3W'—H3WD	109.8
O3B—C4B—C5B	124.3 (3)	H3WC—C3W'—H3WD	108.2
C3B—C4B—C5B	119.0 (3)	C5W'—C4W'—C3W'	106.2 (14)
C4B—C5B—C6B	118.0 (3)	C5W'—C4W'—H4WC	110.5
C4B—C5B—H5BA	121.0	C3W'—C4W'—H4WC	110.5
C6B—C5B—H5BA	121.0	C5W'—C4W'—H4WD	110.5
C1B—C6B—C5B	122.8 (3)	C3W'—C4W'—H4WD	110.5
C1B—C6B—H6BA	118.6	H4WC—C4W'—H4WD	108.7
C5B—C6B—H6BA	118.6	C6W'—C5W'—C4W'	107.0 (12)
O1B—C7B—N1B	121.7 (3)	C6W'—C5W'—H5WC	110.3
O1B—C7B—C1B	119.7 (3)	C4W'—C5W'—H5WC	110.3
N1B—C7B—C1B	118.6 (3)	C6W'—C5W'—H5WD	110.3
C13B—C8B—C9B	119.4 (3)	C4W'—C5W'—H5WD	110.3
C13B—C8B—N1B	118.2 (3)	H5WC—C5W'—H5WD	108.6
C9B—C8B—N1B	122.3 (3)	C5W'—C6W'—C1W'	117.1 (14)
C10B—C9B—C8B	120.0 (4)	C5W'—C6W'—H6WC	108.0
C10B—C9B—H9BA	120.0	C1W'—C6W'—H6WC	108.0
C8B—C9B—H9BA	120.0	C5W'—C6W'—H6WD	108.0
C11B—C10B—C9B	119.7 (4)	C1W'—C6W'—H6WD	108.0
C11B—C10B—H10A	120.2	H6WC—C6W'—H6WD	107.3

### Hydrogen-bond geometry (Å, º)

**Table:**

*D*—H···*A*	*D*—H	H···*A*	*D*···*A*	*D*—H···*A*
N2*A*—H2*AA*···O1*B*	0.86	2.25	3.082 (4)	164
N2*B*—H2*BA*···O1*A*	0.86	2.26	3.091 (5)	163
N1*A*—H1*AA*···O2*B*^i^	0.86	2.28	3.040 (4)	147
N1*B*—H1*BA*···O2*A*^ii^	0.86	2.33	3.077 (4)	145

Symmetry codes: (i) *x*, −*y*+1, *z*−1/2; (ii) *x*, −*y*+1, *z*+1/2.
